# A Focused Ethnography of a 2S/LGBTQIA+ Liaison Nurse Role in Acute Care: Advancing Equity Through Relational Practice

**DOI:** 10.1111/nin.70165

**Published:** 2026-09-27

**Authors:** Ingrid Handlovsky, Allie Slemon, Coady Babin, Sage Schmied

**Affiliations:** ^1^ School of Nursing University of Victoria Victoria British Columbia Canada; ^2^ Institute on Aging & Lifelong Health University of Victoria Victoria British Columbia Canada

**Keywords:** 2S/LGBTQIA+, focused ethnography, health equity, health systems, nursing, safe and affirming care

## Abstract

Two‐Spirit, lesbian, gay, bisexual, transgender, queer, intersex, asexual, and other gender and sexual orientation diverse (2S/LGBTQIA + ) people experience profound interpersonal and structural discrimination in health care settings, contributing to poor health outcomes. This study reports on a novel Liaison Nurse role enacted in a Canadian acute care setting to support equitable care. A focused ethnography was conducted over 7 months, utilizing observations of the Liaison Nurse across two acute care hospitals. Field notes were analyzed using iterative coding and constant comparison to examine institutional considerations and practice dynamics. Three interrelated dimensions of practice were identified via analysis: being present, knowledge of physiologic considerations for 2S/LGBTQIA+ clients, and advocating for the needs of 2S/LGBTQIA+ clients in the health system. These dimensions of practice facilitated the Liaison Nurse's capacity to foster trust, inform clinical decision‐making, and navigate institutional constraints to promote equitable care. The findings highlight that the integration of nursing knowledge and lived experience is central to addressing individual and structural inequities. The Liaison Nurse role functions as a relational, clinical, and systems‐level intervention that advances equity for 2S/LGBTQIA+ people in acute care. Implementing such roles within health systems supports more responsive, inclusive, and equitable care practices.

## 1 Introduction

Over the past several decades, two‐Spirit, lesbian, gay, bisexual transgender, queer, intersex, asexual, and other gender and sexual orientation diverse (2S/LGBTQIA + ) people and allies have successfully advocated for the 2S/LGBTQIA+ rights and freedoms in Canada. Key exemplars of the progress made to protect the rights and, ultimately, health and well‐being of 2S/LGBTQIA+ people include partner visitation rights for gay men hospitalized with HIV/AIDS during the pre‐HAART^1^ era (Hindmarch et al. 2018), legalization of gay marriage (Government of Canada 2015), and legislation prohibiting discrimination targeted at anyone by virtue of sexual or gender identity (Government of Canada 2024). More recently, however, such progress upholding equity and human rights has been offset by growing discrimination targeted at 2S/LGBTQIA+ people including the removal of rainbow sidewalks in some municipalities (Johnson 2024), legislation that dictates that teachers are no longer required to use a student's preferred pronouns (Contact 2023; Latimer 2023), and some regions endeavoring to ban gender affirming care^2^ (Tran 2024).

Health service settings, in particular, are pervasive sites of discrimination targeted at 2S/LGBTQIA+ people, manifesting at interpersonal (e.g., withheld care, inappropriate diagnosis and treatment) and structural (e.g., absence of gender‐affirming care, and barriers to partner visitation) levels (Logie et al. 2019; Schwab et al. 2024), the intersections of which lead to reticence and fear among 2S/LGBTQIA+ people to seek health care due to concerns about their safety. Recent literature highlights that 2S/LGBTQIA+ people are less likely to have a primary care provider (i.e., family physician or nurse practitioner) (Aleshire et al. 2019; Gahagan and Subirana‐Malaret 2018), are less likely to access the emergency room for support with a health issue (Haider et al. 2017; E. B. Quinn et al. 2023), and are more likely to report having experienced a negative interaction with a health provider (McNeill et al. 2023; G. P. Quinn et al. 2015) when compared with heterosexual and cisgender people. The consequences of reduced or absent access to care contribute to inequities in health and health care, ultimately culminating in poorer health outcomes within the 2S/LGBTQIA+ community (Hegazi and Pakianathan 2018; Salway et al. 2022; Schreiber et al. 2021). For example, 2S/LGBTQIA+ people have higher rates of suicide, mental health challenges, and sexually transmitted and blood‐borne infections (STBBIs) (Austin et al. 2022; Lee et al. 2017; Logie et al. 2019; Pinna et al. 2022; Van Gerwen et al. 2020). Resultantly, 2S/LGBTQIA+ people face greater overall mortality compared with heterosexual and cisgender people (Cochran and Mays 2015; Salway et al. 2022).

In response to persistent and widening inequities in health and health care experienced by 2S/LGBTQIA+ people, targeted initiatives have been implemented to address both interpersonal and structural forms of discrimination within health service settings (Goldfarb et al. (2025); Klein and Nakhai 2016). These initiatives include the introduction of formal anti‐discrimination policies, which are visibly displayed throughout health care environments, as well as the use of symbolic markers—such as rainbow flags—to signal inclusivity and safety for 2S/LGBTQIA+ patients (Korbey et al. 2025; Kraus and Duhamel 2018). In addition, some health settings have implemented training initiatives for health professionals, particularly physicians and nurses, aimed at enhancing competency in addressing the specific health needs and experiences of 2S/LGBTQIA+ communities (Klein and Nakhai 2016; Landry 2017). One specific case introduced a novel 2S/LGBTQIA+ Liaison Nurse (LN) role (hereafter referred to as the LN) implemented at an acute care center in Canada. Of note is that the naming of this role was through consultation with Health Authority leaders and community members including Two‐Spirit representation. Based on the findings of a recently conducted scoping review (Slemon, forthcoming) this role represented the first of its kind globally. The purpose of the LN role was to cultivate safe and equitable health care experiences for 2S/LGBTQIA+ patients by providing clinical consultations, supports, referrals, resources, and advocacy. This nascent role was underway for 2 years, with the LN supporting 2S/LGBTQIA+ patients across various health care spaces, including the emergency department, inpatient mental health settings, and medical units. Our research team conducted a comprehensive mixed‐methods study to capture its impacts for 2S/LGBTQIA+ patients and health care providers. This article reports on the qualitative component of the study, for which the research aim was to capture the essential elements of how the LN role unfolds within everyday interactions with 2S/LGBTQIA+ people seeking health care, and describe the broader social and cultural processes inherent to the health setting that influence the role.

## 2 Methods

### 2.1 Theoretical and Methodological Foundations

This investigation was conceptually informed by Farmer's health equity framework that highlights how social structures systemically disadvantage equity‐owed groups by perpetuating the very harms that disadvantage them (De Maio 2015; Farmer 2004). Within this framing, health equity is understood as fundamentally structural, reflecting how the social conditions that contribute to disadvantage for equity‐owed groups are produced and sustained through the political and economic organization of the social world (De Maio 2015; Farmer 2004). Further, we drew on Doane and Varcoe (2007) relational inquiry approach that posits nursing practice as a key means to advancing health equity by redressing processes that contribute to disadvantage and upholding equity within health and social systems. Drawing on the combined approaches of Farmer and Doane and Varcoe foregrounds health inequities experienced by 2S/LGBTQIA+ people as rooted in interpersonal and structural discrimination, while highlighting the capacity of nursing practice to advance equity through reflexive, relational, and ethically responsive everyday care.

Methodologically, we employed a focused ethnography approach increasingly taken up in health disciplines to explore the social and cultural processes that shape how health care provision is delivered and experienced (Baumbusch et al. 2018; Trundle and Phillips 2023). In contemporary focused ethnography, culture refers to the specific practices and interactions embedded within modern, complex systems such as health settings (Cruz and Higginbottom 2013). This conceptualization represents a shift from earlier anthropological uses of the term, which often framed culture as homogeneous societies informed by colonial perspectives that positioned non‐Western groups as inferior to the colonizer (Muecke 1994). Focused ethnography has been described as ethnography on a smaller scale to emphasize the time‐bounded approach adopted by this methodology and its specific and tailored focus on a particular element of health care (Trundle and Phillips 2023). Applied to this project, focused ethnography enabled us to investigate a specific practice (i.e., a nursing role) within a bounded setting and defined time‐period, using intensive and targeted data collection techniques to achieve depth of exploration (Trundle and Phillips 2023). Specifically, central key tenets of focused ethnography—nuance and context—were key to this study (Baumbusch et al. 2018; Trundle and Phillips 2023). *Nuance*‐oriented inquiry to capturing everyday elements of the LN role that are challenging to articulate yet essential for ensuring a meaningful and effective health care encounter. *Context*‐guided examination of the social and cultural processes that inherently shape the LN role and hold great implications for how the role is practiced.

Our research team comprises health researchers—including Registered Nurses—with clinical expertise spanning a range of practice settings and contexts. In addition, our team holds representation from 2S/LGBTQIA+ communities and allied positions. The diverse intersecting identities and social locations of this team guide our commitment to upholding equity for 2S/LGBTQIA+ people in health settings.

### 2.2 Setting

The 2S/LGBTQIA+ LN role was implemented in early 2024, with one 2S/LGBTQIA+ LN offering direct support to 2S/LGBTQIA+ clients across two Victoria, BC hospitals. Both hospitals offer health care across a range of acute settings, including emergency, medical/surgical, mental health, and specialty care units. In this context, the 2S/LGBTQIA+ LN role—envisioned as a position for a Registered Nurse—was designed to offer direct client care across each hospital, with both clients and health care providers able to initiate referrals to this service. The individual working in this role was a trans woman who had played a central role in envisioning and advocating for the implementation of the role and brought a wealth of experience in gender‐affirming care, mental health, and emergency care.

### 2.3 Ethical Considerations

This study received ethical approval from Island Health and UVic, UBC, and Simon Fraser ethical review boards. Upon each encounter within the health setting, the LN would inform the individual (i.e., client, family member, health professional, etc.) that there is a research study being conducted, and that a researcher will be recording field notes pertaining to the interaction. Though this did not occur in practice, our identified process was that if any person expressed discomfort or articulated they did not wish to have field notes recorded, the researcher would remain outside the space until the interaction with the LN had concluded. During the recording of field notes, researchers focused predominantly on the LN actions and practices, and any information relating to other individuals and the milieu of the LN was de‐identified to ensure privacy and confidentiality. The LN provided consent for this study with acknowledgment that despite the possibility that they could be identified, the data pertained to the work specifically and not to personally identifying details.

### 2.4 Data Collection

Data were collected over a 7‐month period throughout 2024–2025 and comprised qualitative observations alongside the 2S/LGBTQIA+ LN, to capture nuanced aspects of the everyday work associated with this role in an in‐depth manner. Everyday work represents an analytical lens in the context of this study, referring to the socially organized activities, interactions and responsibilities enacted by the 2S/LGBTQIA+ nurse in enacting care (Baumbusch et al. 2018). Further, everyday work is situated within institutional dynamics, which refer to the socially organized, institutional, and relational processes that are negotiated by the actors embedded in these contexts (Trundle and Phillips 2023). A “shadowing” approach to observations was utilized, as per qualitative ethnographic approaches (McDonald 2005), where a member of our research team followed the 2S/LGBTQIA+ LN to capture all aspects of the everyday work ranging from a few hours to a full shift. Shadowing involved the researcher being present during encounters with clients and their visitors (with verbal consent) observing interactions with other health care providers, and attending meetings or sessions in which the 2S/LGBTQIA+ LN participated or took a leading role. Observation sessions were scheduled as per LN and researcher availability. Throughout observations, the researcher recorded brief notes commonly referred to as *jottings* (Trundle and Phillips 2023) to capture salient moments, key language used by the 2S/LGBTQIA+ LN, and other elements. Following each shadowing session, the researcher developed a comprehensive field note documenting both *what* occurred and *how* the day's events illuminated core dimensions of the 2S/LGBTQIA+ LN role. A field note guide supported this process by prompting reflection on observations and impressions, including: *What characterized the encounters between the LN and clients? How was the LN received? How does this specific health care context shape what the LN is doing and how they are doing it?* And *how does what the LN is doing shape the health care context?* The guide also included space for general observations of the setting, reflexive notes, and additional comments. Observation selection, organization, and interpretation were shaped by the study aim, the bounded context, and an iterative analytic process. Observations were purposively directed toward routine and situational practices relevant to the nursing role, structured around key interactions, activities, and workflows.

### 2.5 Data Analysis

The field notes, which constituted data, were treated not as neutral records, but as situated accounts of observed practices, interactions, and health setting context. Analysis commenced following completion of data collection and began with a broad, thorough reading of the field notes to develop familiarity with the dataset as a whole and to allow for initial reflections (Sandelowski 2010). The next step was a closer read of the field notes, guided by three broad concepts aligned with the research aims to support a deeper examination of: (1) the manner of interactions the LN had with 2S/LGBTQIA+ people seeking care; (2) how the LN's work was shaped or constrained by the dynamics of the health setting; and (3) the potential impacts of the LN's work on health setting dynamics. With the three domains serving as a scaffold, focused coding proceeded where field notes were analyzed line by line and patterns of experience were grouped into codes such as “body language,” “identity considerations,” and “upholding client autonomy” (Charmaz 2014; Sandelowski 2010). Following code development, the data were primarily analyzed by the first author.

Once codes were established, the process of constant comparison facilitated comparing and contrasting codes (Charmaz 2017). As constant comparison progressed, through iterative coding and constant comparison across observations, field notes were progressively developed into analytical insights pertaining to recurring patterns, meanings, and tensions in the nursing role and the setting in which it was enacted. Here, constant comparison enabled comparison of codes across situations and time points to identify recurrent practices and contextual variation within a bounded setting to support exploration of the nursing role situated within health system dynamics. Reflexivity was integral to the analytic process and required each team member systematically reflect on their social locations and the potential influence of assumptions and biases (Sandelowski 2010). Consequently, reflexivity was operationalized through team dialog—comprising sharing of reflections, questions and analytic insights—across the entirely of the research process from project conceptualization to data collection, analysis and writing. Further, informal interviews (*n* = 4) were conducted with the 2S/LGBTQIA+ LN as a form of member checking, in which preliminary insights were shared to ensure accurate representation of insights. Engaging in this kind of member checking was salient in the context of an institutional ethnography where more commonly observed means of member checking—such as sharing findings with interview participants, as noted across many qualitative approaches, for example—is not possible.

## 3 Findings

A total of 17 discrete observation sessions were conducted, ranging 2–10 h per session for a total of 83 observation hours. Because observation sessions comprised part of, or at times an entire nursing shift, a variety of interactions, encounters, and activities were captured. Through iterative coding and constant comparison of field notes, we came to understand that the LN role encompasses numerous strategic and deliberate practices to support 2S/LsGBTQIA+ people within acute health care settings. These practices include relational engagement approaches and a broad range of psychosocial, logistical, advocacy, and direct care supports. Our findings highlighted that grounding the role in the discipline of nursing prioritizes communication and relational engagement to provide a powerful support for 2S/LGBTQIA+ people, many of whom have experienced discrimination within institutional contexts. Recognition of the immensity of the supportive element of the LN role is effectively captured as follows:

She said something that stayed with me: “The person in front of me is telling me what will make them more comfortable, and that's what I can provide.”

– Observation 13

In addition to the centrality of relational engagement to the LN role, the LN's advanced assessment skills and physiological expertise intersected with their lived experience as a 2S/LGBTQIA+ community member, enabling a unique integration of experiential and clinical knowledge. This intersection of clinical expertise and lived experience informed therapeutic conversations and supported health care providers' decision‐making in ways that attended to the distinct needs and considerations of 2S/LGBTQIA+ people. Our observations further illustrated the realities of institutional dynamics, structures, and processes on the work of the LN, while simultaneously highlighting the LN's capacity to meaningfully influence and shift these conditions. Collectively, these findings demonstrate that the LN brings a comprehensive and integrated skill set that enables effective and meaningful support for 2S/LGBTQIA+ people as they navigate health care encounters. Ultimately, we identified three dimensions of practice, described below, alongside the key contextual dynamics that shape how the LN role is enacted and how the LN practices, in turn, influence institutional processes: being present, knowledge of physiologic considerations for 2S/LGBTQIA+ clients, and advocating for the needs of 2S/LGBTQIA+ clients in the health system.

### 3.1 Being Present

A persistent theme across observations was the palpable presence the LN cultivated when interacting with clients. By drawing on a repository of nursing skills, the LN was able to consider the situation at hand, namely, gauging the psychosocial state of the client and determining the most appropriate approach to establishing comfort and safety in the context of what for many were unfamiliar and uncomfortable circumstances. The LN's capacity for presence was highly influential, as she fostered comfort and safety for clients navigating complex health and/or social challenges through attentive body language, the encouragement of dialog about fears and frustrations, and the validation of client's expressed feelings. The LN, through strategic interpersonal approaches, was able to cultivate an environment of validation and support and in doing so garnered trusting relationships with clients:

She used active listening techniques including summarizing, reflections, and open‐ended questions…the patient fist bumped the LN and said ‘good to see you’ when saying goodbye, a small gesture that spoke volumes about the rapport the LN has with this client.

– Observation 2

Approaching the interaction in a way that demonstrated undivided attention and genuine investment in ensuring the client felt heard and validated served to cultivate a degree of trust and comfort. The positive regard the client felt for the LN was evident in how she was received by the client, and the integration of the friendly gesture of a fist bump to diffuse the professionalism of the environment and introduce a degree of warmth and friendliness to the encounter. Such seemingly small acts to generate comfort were understood to have a marked impact on the development of positive rapport with clients.

The positive regard the LN cultivated with clients was largely attributable to her intentional prioritization of the human experience over task completion. By creating space to engage with clients as whole persons, the LN not only enhanced clients' experiences of care but also strengthened her capacity to deliver comprehensive and responsive support. The development of trusting relationships fostered a relational environment in which clients felt more comfortable asking questions, seeking clarification about processes and procedures, and voicing concerns or reservations. Having access to this relational and contextual information positioned the LN to identify, tailor, and propose appropriate supports aligned with client needs and readiness to receive care:

A particularly meaningful moment was watching her interaction with a [patient] with OCD and treatment resistance. He was reluctant to engage in outpatient treatment programs, and offered many reasons to not engage in therapies… The LN approached the situation with empathy and creativity, offering options like queer‐specific housing and support groups, and emphasizing the importance of queer community and outpatient mental health support. This interaction demonstrated the Liaison Nurse's ability to meet patients where they are at, without judgment, and collaboratively develop a plan that prioritized safety and comfort.

–Observation 5

Despite the LN's commitment to being present, the reality that systemic barriers are a persistent challenge was evident throughout observations. Namely, the work of being present is greatly constrained by resource insufficiency, specifically, the volume of clients to be seen, and the diversity of supports that are required for clients exceeds what is reasonable for one person to provide. The juxtaposition of the risk management approach often frequented in health care versus the person‐centered and preventative nature of the LN's work was emphasized:

We discussed how person‐centered the nurse is (and I saw this in action with a client) and how the [hospital] environment does not lend itself to this. *–* Observation 1

The LN's expression of misalignment between person‐centered approaches to supporting clients and the realities of contemporary health systems encompassed a diversity of challenges. The demands on health care providers due to staff shortages and complex health issues in the wake of a post‐COVID system coupled with processes and practices that do not consider the unique needs of 2S/LGBTQIA+ people introduced obstacles for the LN to truly support clients in the most meaningful way possible. Together, these structural constraints introduced significant obstacles to the LN's ability to support clients in ways that were experienced as both meaningful and effective, underscoring the broader incompatibilities between equity‐informed, relational nursing practice and contemporary acute care environments.

### 3.2 Knowledge of Physiologic Considerations for 2S/LGBTQIA+ Clients

The work of the LN extended beyond meaningful relational engagement with clients to encompass advanced biomedical knowledge specific to the medication, treatment, and care considerations relevant to 2S/LGBTQIA+ populations. This specialized knowledge is critical for ensuring that clients receive care that is not only clinically appropriate but also responsive to the unique physiological and treatment‐related needs that may accompany diverse gender identities, sexual orientations, and embodiment experiences. The LN's expertise included a nuanced understanding of medication management, potential drug interactions, and treatment implications such as those related to hormone therapies, surgical histories, and co‐occurring health conditions that are frequently under‐addressed within general clinical practice:

She asked about her (the client) experience taking estrogen, if she had any side effects, and if she had any specific goals regarding HRT/GAC [hormone replacement therapy/gender‐affirming care], to which the patient explained she was looking for breast growth and bottom surgery… she also looked at the patient's most recent blood work to assess her estrogen and testosterone levels while on estradiol and spironolactone, and indicated that she could be on a higher dose of estradiol, which she planned to communicate to the patient's doctor.

‐Observation 8

Here, the LN was able to apply complex and specialized knowledge related to physiological considerations in a unique clinical circumstance and therefore ensure that the client was adequately supported. The specific health needs of 2S/LGBTQIA+ clients frequently require a level of clinical expertise that extends beyond general practice, and the LN was uniquely positioned to provide this depth of knowledge. Possessing advanced clinical competence, the LN further played a pivotal role in supporting the work of other health care providers—most notably physicians—by contributing expertise that informed safe, appropriate, and effective care for 2S/LGBTQIA+ clients. A key facet of the LN's everyday work involved consultation and education with health care providers regarding the specific physiological and treatment‐related needs of 2S/LGBTQIA+ populations. It was consistently observed that many physicians lacked advanced training in this area, particularly with respect to the care of transgender patients. Exemplars included assessing hormone therapy in the context of acute medical presentations, as well as supporting comprehensive care planning following gender‐affirming surgeries. In these contexts, the LN functioned as a critical clinical resource, ensuring that physicians were equipped with the knowledge necessary to deliver care that was both clinically sound and responsive to the unique needs of 2S/LGBTQIA+ patients:

The client moved to the island from [region] and recently sought care at [hospital] for complications related to their vaginoplasty. The provider at [hospital] was not trained in pelvic assessments for people with a vaginoplasty. The [region] provider reached out to the LN to ask if she could do an exam inclusive of the pelvic region for the client and report her assessment to the physician.

– Observation 16

In this instance, the LN worked collaboratively with other members of the care team to ensure that an essential clinical assessment—one the attending physician did not feel adequately prepared or comfortable to perform—was completed. By assuming responsibility for this assessment, the LN ensured that care was both safe and clinically appropriate for the client. The LN also played a key role in synthesizing and clearly communicating salient clinical findings to the broader care team, thereby supporting timely and informed decision‐making.

The formal inclusion of lived experience as a component of the LN role, as outlined in the job description filed with HEABC, allowed for intentional opportunities for the LN to draw upon the productive intersection of experiential knowledge and nursing expertise. Explicit embedding of lived experience in the role description legitimized lived experience as a form of knowledge that meaningfully informs clinical practice, rather than positioning it as ancillary or informal. The LN's lived experience as a trans woman offered distinct and valuable possibilities for enhancing patient care by informing relational engagement, clinical judgment, and advocacy efforts. Through drawing on her own experience as a transgender woman, the LN was able to anticipate potential points of vulnerability that may otherwise go unrecognized, and engage with clients in ways that fostered trust, safety, and mutual understanding:

Not only did the LN identify concerning patterns of disordered eating and signs of body dysmorphia, but she was then able to explore these concerns through the lens of trans identity and hormone therapy…this was a powerful example of how the LN, with both a nursing/medical background and extensive knowledge of trans lived experiences, can uniquely support trans clients.

– Observation 9

The LN holding lived experience as a trans person enhanced her clinical capacity to deliver equity‐oriented, affirming, and contextually informed care. Approaching the scenario from a position of shared community situatedness supported the LN's ability to establish trust when initiating discussion of a sensitive issue—disordered eating—further complicated by considerations related to hormone therapy. By integrating clinical expertise with lived experience, the LN was able to respond with compassion, insight, and effectiveness, thereby ensuring that the client was optimally supported.

### 3.3 Advocating for the Needs of 2S/LGBTQIA+ Clients in the Health System

A substantial facet of the daily work of the LN nurse was recognized as identifying and advocating for the needs of 2S/LBTQIA+ clients, which comprised a wide range of activities from supporting health professionals' engagement approaches to creative opportunities to bolster patient confidence and comfort to administrative considerations. Given that many 2S/LGBTQIA+ people have endured unpleasant and/or harmful experiences within health settings, the advocacy component of the LN represents a key facet of her work that contributed to enhanced client experiences. The LN, as a member of the 2S/LGBTQIA+ community, introduces an element of comfort that clients respond to, feeling enhanced comfort with someone who understands their concerns through the lens of lived experience. Through advocating for clients, the LN seeks to acknowledge and address the discomfort a client may be experiencing and through doing so serves to even slightly alleviate anxiety and/or discomfort:

The Liaison Nurse immediately recognized the patient's discomfort caused by excessive supervision; there were 3 staff members in the room… she requested staff to leave the room for our conversation, and prioritized creating a safe, supportive environment…The Liaison Nurse's actions and conversation upheld the patient's autonomy and dignity, creating a foundation of trust that is important for ongoing support.

– Observation 6

The advocacy undertaken by the LN encompassed a continuum of actions, ranging from subtle recommendations that enhance client comfort to more substantive procedural interventions aimed at upholding identity and dignity. The health system was observed to operate in ways that neither prioritizes nor consistently situates care within gender‐affirming practices. For instance, processes related to the collection and storage of personal information carry significant implications for the safety and comfort of 2S/LGBTQIA+ individuals. As illustrated in the observation below, the privileging of legal names over preferred names is embedded within institutional protocols and may result in profound psychological harm for clients:

In one instance, the LN advocated for the use of a client's preferred name, which led to the staff nurse explaining ED staff would use the client's preferred name, except during lab work and imaging, when staff are legally required to confirm the name on the health card. At one point, while attempting to justify the ED's use of clients' dead names^3^, the RN said, ‘we don't do this to be mean.’

– Observation 14

In response to systemic challenges, the LN engages in advocacy that extends beyond addressing structural considerations—such as how personal information is collected and stored—to include educational efforts that support health care providers in aligning their practices with gender‐affirming principles. Through this advocacy, the LN also facilitates knowledge translation among other health care providers, many of whom may be unaware of the challenges experienced by 2S/LGBTQIA+ clients within the health system. As a result, providers may inadvertently overlook the importance of approaches that mitigate anxiety and discomfort; the LN's role is therefore instrumental in fostering more responsive and affirming care practices.

The knowledge of health care systems that the LN possesses, combined with her lived experienced as an 2S/LGBTQIA+ person, positioned her such that she could effectively circumvent systemic barriers to encourage providers to shift practices to support a gender‐affirming approach. At times, this required acknowledgment of system requirements whilst recognition of how to subvert these parameters such that client safety and comfort were ensured:

She [the LN] explained that the RN had shared incorrect information and that two forms of legal identification are required, but neither must include a specific name. The LN noted that she would follow up with the RN to suggest alternative ways of approaching conversations about pronouns and preferred names with trans and gender‐diverse clients.

– Observation 14

In this instance, the LN's distinctive knowledge derived from the intersection of advanced nursing knowledge and lived experience creates substantial opportunities to support other health care providers in more effectively addressing the health needs of 2S/LGBTQIA+ individuals. In this capacity, the LN serves as a critical resource in care provision, not only through consultation on complex clinical situations requiring nuanced physiological understanding, but also by attending to the psychosocial dimensions of individuals' experiences within the health system. By embedding advocacy into practice and fostering its uptake among other providers, the LN generates a ripple effect that can shift institutional dynamics and improve care for 2S/LGBTQIA+ individuals in diverse and sustained ways.

The three identified dimensions of practice illustrate that the LN role functions as an equity‐oriented nursing intervention that integrates relational engagement, specialized clinical expertise, and advocacy to support 2S/LGBTQIA+ people in acute care. Central to the role is the LN's capacity for presence and relationship‐building, fostering trust and safety within institutional contexts often experienced as marginalizing. Advanced nursing assessment skills, combined with expertise in gender‐affirming and physiologic care and formally recognized lived experience, position the LN as a critical clinical and relational resource. The advocacy systematically employed by the LN highlights the structural constraints that persist whilst illuminating how the actions of the LN serve to shift institutional dynamics to uphold equity for 2S/LGBTQIA+ people.

## 4 Discussion

This exploration provides valuable insight into the utility of a dedicated health provider role situated in the discipline of nursing and informed by lived experience to address the diverse and intersecting needs of 2S/LGBTQIA+ people within an acute care context. Given the extent of interpersonal and structural discrimination endured by 2S/LGBTQIA+ people in health settings (Handlovsky et al. 2018; Kia et al. 2024; Kraus and Duhamel 2018; Reynolds 2020), the need for salient approaches to cultivate safe and respectful encounters is timely and necessary. Improving health care experiences can shift perceptions of the health system and, in turn, foster improved trust and engagement among 2S/LGBTQIA+ people when health services are needed—an essential condition for achieving and sustaining positive health outcomes (Broholm et al. 2023; Pang et al. 2025; Salway et al. 2018, 2022). It is well substantiated that system dynamics influence role uptake, utility, and efficacy and thus must be considered to ensure health experiences and outcomes are optimal (Bauer et al. 2015; Baumann and Cabassa 2020). Through the application of a focused ethnographic approach, we were able to capture institutional considerations via two key facets: how broader systemic and institutional dynamics shape the positioning and everyday practices of the LN, and the ways in which the LN actively navigates, negotiates, and intervenes within these systems to advocate for more affirming, equitable, and responsive care for 2S/LGBTQIA+ people. By foregrounding the relational, contextual, and practice‐based dimensions of the LN role, the findings of this exploration emphasize how such positions can function as both clinical and systems‐level interventions. This is a particularly powerful finding amidst the current health care climate in Canada, where regional health settings are undergoing extensive restructuring (Johnson 2025; PHSA 2025). As such, the need for innovative and streamlined approaches to meet care needs are necessary and integral to ensuring the unique needs of equity‐owed groups such as 2S/LGBTQIA+ people are upheld amidst system upheaval and change.

Given the extent of disadvantage that 2S/LGBTQIA+ people experience in health systems, it is unsurprising that our findings underscore the profound importance of presence to the work of the LN, demonstrated through practices such as attentive body language, validation of concerns, and responsiveness to questions. The importance of presence as a key form of support for 2S/LGBTQIA+ people in acute care settings is twofold, reflecting both the inherent vulnerability associated with health care encounters and the disproportionate adversity 2S/LGBTQIA+ people often experience when seeking care (Kennedy et al. 2024; Kuzma et al. 2019; Salway et al. 2018). The immensity of presence to the LN role also substantiates its positioning within the fundamentally relational discipline of nursing. Relational practice is identified as a core competency for the nursing discipline, rooted in the understanding that nursing practice is constituted through relationships with individuals, communities, and institutions (Doane and Varcoe 2007 2020; Thorne 2015). The practice of nursing necessitates extending beyond basic interpersonal skills toward a nuanced and contextual understanding of people to meaningfully support them through experiences of health and illness as a relational process of *being with* in addition to *doing* (Doane and Varcoe 2007 2020; Thorne 2015). By embodying and enacting relational practice, nurses can meaningfully and effectively address injustice and uphold equity for 2S/LGBTQIA+ people within acute health care settings (Abu 2020; Thorne 2015).

The work of this investigation served to highlight the persistent gaps in clinician knowledge related to the unique physiological considerations of 2S/LGBTQIA+ people, a phenomenon well documented in the literature (Bristol et al. 2018; Freaney et al. 2024; G. P. Quinn et al. 2015). Our observations reveal the often‐complex physiological needs of transgender people that many clinicians feel ill‐equipped to manage, which warrants educational support inclusive to theory and practice‐based education. The need for accessible, comprehensive, and up‐to‐date education—especially related to hormone replacement therapy and pre‐ and post‐operative care for transgender people—emerged as a critical finding and is echoed in existing research (Cheung et al. 2019; Ker et al. 2020; Shuster (2016). Our findings further enforce the need for integration of transgender health guidelines into health professional training—specifically physicians, nurse practitioners and nurses. Embedding salient transgender health considerations within health professional curricula ensures that practitioners enter practice with this foundational knowledge, which can then be further strengthened and updated through lifelong learning opportunities. for example, health settings are well positioned to provide streamlined and evidence‐informed training for clinical staff to support consistent and effective care. The efficacy of such programs is noted in the literature in relation to supporting health provider proficiency to meaningfully and effectively meet the needs of 2S/LGBTQIA+ people (Damery et al. 2025; Klein and Nakhai 2016; Stokes and Lecuyer 2023). Such educational opportunities are well situated in the context of health settings they can be tailored to the unique contextual dynamics of each setting and can be offered in a variety of flexible formats (lunch and learn, toolkits, webinars, and so forth).

The findings also illuminate the centrality of advocacy to the LN role, particularly in advancing institutional processes that uphold dignity, respect, and equity for 2S/LGBTQIA+ people. The LN was uniquely positioned to identify and propose alternative approaches to navigating system‐level processes in ways that mitigated harm and promoted inclusive care. Notably, the intersection of nursing knowledge and lived experience enabled the LN to recognize how routine health system practices—such as the collection and storage of personal information—can unintentionally contribute to harm for 2S/LGBTQIA+ people, often in ways that remain invisible to other health care providers. These findings reinforce the value of bottom‐up approaches to system‐level change, wherein advocacy, education, and relational support embedded within a dedicated role can effectively shift institutional dynamics (Kraus and Duhamel 2018; Shatrov et al. 2021; Van Steenis et al. 2025; Vazquez‐Calatayud et al. 2024). Such approaches offer immediate and meaningful support while the process of broader structural reform—often requiring significant time and coordination—is conceptualized and implemented (Kraus and Duhamel 2018; Shatrov et al. 2021). Indeed, “bottom‐up” approaches align with grassroots and community‐based approaches that 2S/LGBTQIA+ communities have effectively operationalized to resist discrimination and improve health and well‐being (Goodyear 2024; Moseley et al. 2008; Scheadler et al. 2023). Tactics that support successfully navigating challenges and meeting needs include consultation, dialog, and collaboration, centering the voices of identified groups in defining both their needs and the most meaningful ways to meet them (Belda‐Miquel et al. 2020; Roche 2017). Further, the utility of the LN role to address systems‐level interventions offers insights for informing the intentional and sustainable integration of the LN role within health care environments, given the demonstrated potential of the role to disrupt normative practices, influence institutional cultures, and improve care experiences and, by extension, health outcomes for 2S/LGBTQIA+ people.

There are several limitations inherent to this investigation that must be noted. The focused ethnographic approach enabled nuanced consideration of how the role is practiced—including institutional constraints and opportunities for the LN to likewise alter institutional dynamics—on one particular setting. As such, the findings may have limited applicability to other acute care settings, particularly those with differing organizational or structural designs. The need for explorations of a similar nature pertaining to the LN role in additional acute care settings and more broadly to community contexts, for example, would provide key information about the scope and implications for the role to support the health and well‐being of 2S/LGBTQIA+ people. Further, although this exploration captured essential information about how the LN role is practiced in a particular health setting, and observations that illuminated how the LN was received by 2S/LGBTQIA+ clients, we did not explicitly collect experiences and perspectives of 2S/LGBTQIA+ people who received care from the LN. Eliciting the perspectives of care recipients would offer comprehensive information to identify elements of the role that were deemed particularly beneficial (and those that were not) to improve the role to effectively support the health and well‐being of 2S/LGBTQIA+ people.

## 5 Conclusion

This investigation provides insights into a novel LN role dedicated to supporting health encounters of 2S/LGBTQIA+ people in a specific health setting. This role, to our knowledge, is the first of its kind, and our findings position the LN role as a relational, clinical, and political intervention that confronts inequities endured by 2S/LGBTQIA+ people that are perpetuated within health systems. By mobilizing advanced nursing knowledge, advocacy, and formally recognizing lived experience, the LN enacts equity‐oriented practice within institutional contexts that frequently discipline, constrain, and normalize exclusion. The findings suggest that such roles do more than improve individual care experiences but serve to expose and disrupt taken‐for‐granted practices that reproduce harm under the auspice of efficiency. As health systems continue to undergo restructuring, the LN role illuminates nursing's ethical and political capacity to resist inequity through everyday clinical work.

## Author Contributions

**Ingrid Handlovsky:** conceptualization, methodology, data curation, formal analysis, writing‐original draft. **Allie Slemon:** conceptualization, methodology, data curation, formal analysis‐review and editing. **Coady Babin:** formal analysis, writing‐review and editing. **Sage Schmied:** formal analysis, writing‐review and editing.

## Ethics Statement

This study received ethical approval from the University of Victoria and Island Health ethical review boards (H24‐00957).

## Consent

Each participant provided informed, verbal consent.

## Conflicts of Interest

The authors declare no conflicts of interest.

## AI Disclosure

The authors confirm that no generative AI or AI‐assisted technologies were used in the writing, editing, data collection, analysis, or generation of this manuscript.

## Data Availability

The data that support the findings of this study are available on request from the corresponding author. The data are not publicly available due to privacy and ethical restrictions.

## Footnotes

^1^
Pre‐HAART refers to prehighly active retroviral therapy, and marks a time period spanning from the early 1980's to the mid 1990's characterized by the absence of HIV/AIDS treatment and rampant discrimination targeting gay men (Forstein 2013; Shulze 2015).

^2^
Gender‐affirming care refers to health delivery and support by providers who are informed about the unique needs of transgender people, and thus tailor their approaches and practices to reflect knowledge of these needs and to support the person's identity to ensure a comfortable and supportive care encounter (Bhatt et al. 2022).

^3^
A “dead name” refers to the birth name of a transgender person who has since changed their name (i.e., the chosen name) as part of their gender transition (Sinclair‐Palm and Chokly 2023)
